# Changes in maxillary dental arch morphology after implant treatment in the alveolar cleft region

**DOI:** 10.1002/cre2.384

**Published:** 2020-12-20

**Authors:** Kilwoo Ahn, Hitoshi Sato, Yuji Kurihara, Hiroshi Ogura, Tatsuo Shirota

**Affiliations:** ^1^ Department of Oral and Maxillofacial Surgery, School of Dentistry Showa University Tokyo Japan; ^2^ Department of Information Science, Faculty of Arts and Sciences at Fujiyoshida Showa University Tokyo Japan

**Keywords:** alveolar cleft, cleft lip and/or palate, dental arch morphology, dental implant

## Abstract

**Background:**

Few reports have examined dental arch morphology (DAM) after dental implant placement in cleft patients and its actual state is unclear.

**Objective:**

To analyze the presence of changes in DAM and influencing factors in cleft lip and/or palate (CLP) patients who receive implant treatment in the alveolar cleft region.

**Methods:**

Subjects comprised 20 CLP patients in whom maxillary dental arch width (DAW) was evaluated before and after implant treatment based on computed tomography data. First, widths between the canines (W3), between the first premolars (W4), between the second premolars (W5), and between the first molars (W6) were measured before and after surgery. Changes in distance were analyzed using the Wilcoxon signed‐rank test, revealing a significant increase in W6. Analysis of Co‐Variance was performed with the difference in W6 after implant treatment as the response variable, and the following six items as explanatory variables: sex; cleft type; age at alveolar bone graft; time to implantation after bone grafting; number of implants; and time after completion of the observation period.

**Results:**

The reduction in W6 was larger in the order of complete bilateral CLP, complete unilateral CLP, and unilateral cleft lip and alveolus, and the change decreased with an increasing number of implants.

**Conclusions:**

Implant treatment of the alveolar cleft region may result in a slight reduction in width of the dental arch after treatment completion.

## 1 BACKGROUND

Treatment of alveolar cleft by bone grafting followed by the induction of eruption or orthodontic gap closure with permanent teeth adjacent to the alveolar cleft is considered ideal for oral rehabilitation in cleft lip and/or palate (CLP) patients (Amanat & Langdon, 1991; Boyne & Sands, 1976). However, in cases where oral rehabilitation by orthodontic gap closure is problematic due to unilateral missing teeth and the resulting dental asymmetry, dental implants represent a good option that does not burden the teeth adjacent to the alveolar cleft. In CLP patients, due to the effects of scar tissue formed after surgery during childhood, secondary deformities such as recessed midface, dental arch stenosis, dentition congestion, and reverse occlusion are constantly observed (Ishikawa et al., 1998; Marcusson & Paulin, 2004). As implant treatment does not prevent relapse of the dental arch morphology (DAM), unlike a fixed prosthesis would (Caballero et al., 2019), the DAM may lack stability after treatment (Pucciarelli et al., 2020).

To verify the hypothesis that maxillary DAM after dental implant placement in the alveolar cleft and its actual state is unstable, the presence of changes in DAM was analyzed in this study. Moreover, factors influencing DAM in CLP patients who received implant treatment in the alveolar cleft region were analyzed.

## 2 METHODS

### 2.1 Setting and participants

Evaluation of dental arch width (DAW) on computed tomography (CT) images acquired before and after surgery was possible in 20 CLP patients who underwent autogenous iliac cancellous bone grafting to the alveolar cleft, followed by implant treatment of the alveolar cleft region at our university dental hospital between 2009 and 2016. All 20 patients were selected as subjects. This study was approved by the medical ethics committee of our university (DH2017‐003). All study procedures were performed in full compliance with the Declaration of Helsinki or comparable ethical standards.

CT images acquired immediately before implant surgery and at the completion of implant treatment were used. A HiSpeed QX/I x‐ray CT system (GE Healthcare Japan, Tokyo, Japan) was used for acquisition. Acquisition conditions were as follows: tube voltage, 120 kV; tube current, 260 mA; and slice thickness, 1.25 mm.

### 2.2 Analysis of changes in maxillary DAM

**FIGURE 1:**
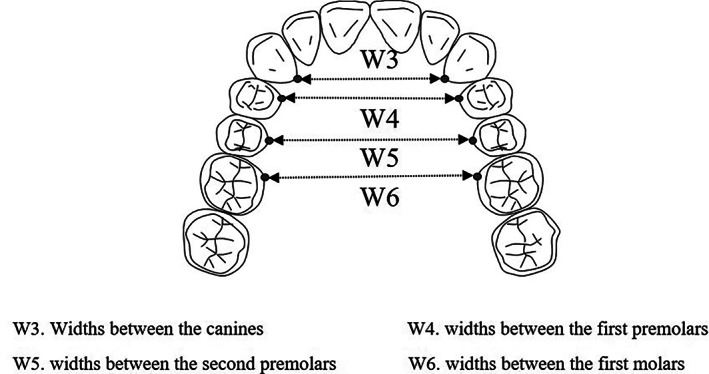
Schema of the dental arch widths (DAWs)

Three‐dimensional images of the maxillary dental arch were constructed from DICOM CT data that were acquired immediately before implant surgery and after the completion of implant treatment using implant‐planning software (Blue Sky Plan; Blue Sky Bio LLC, Grayslake, IL). Measurement points were the cemento‐enamel junctions immediately below the cusps of the canines, and the cemento‐enamel junctions immediately below the mesiolingual cusps of the first and second premolars and first molar. The X, Y, and Z coordinates of each measurement point were determined, and DAW was measured for each region (Figure 1). The same tester measured these three coordinates in each region at 7‐day intervals, and means rounded off to the second decimal place were adopted as measured values.

### 2.3 Statistical analysis

All statistical analyses were performed using SPSS version 22 and R version 3.5.0 software. The level of significance was set at <5%. When the sample size is 20 subjects, the power of the Wilcoxon signed‐rank test would be 0.8 (before vs. after treatment).

#### 2.3.1 Measurement accuracy of analyses

Using a method to expand the Wilcoxon signed‐rank test for multiple comparisons, we analyzed whether differences were present among the 1st, 2nd, and 3rd measured values in each region. The null hypothesis was that population means of the 1st, 2nd, and 3rd measured values would be equal. The Holm method was used for probability adjustment for multiple comparisons.

#### 2.3.2 Analysis of the presence of changes in each DAW over time

Differences in DAW after treatment in the bilateral canine region (W3), first premolar region (W4), second premolar region (W5), and first molar region (W6) were analyzed using the Wilcoxon signed‐rank test. The null hypothesis was that DAWs before and after implant surgery would be the same.

#### 2.3.3 Analysis of factors influencing changes in DAW over time

First, values in measurement regions where the population mean was considered different after implant treatment were measured three times and mean values were calculated. The difference in mean after treatment was calculated, and analysis of covariance was performed with this value regarded as a response variable and the following six items regarded as explanatory variables: sex; cleft type; age at bone graft to the alveolar cleft region; time to implant treatment after bone grafting; number of implants; and observation period.

## 3 RESULTS

### 3.1 Clinical results

Background characteristics of the 20 patients (9 males, 11 females) were as follows: cleft lip and alveolus (CLA), *n* = 1, (1 male); unilateral CLP (UCLP), *n* = 11 (5 males, 6 females); bilateral CLP (BCLP), *n* = 8 (3 males, 5 females). Occlusion in these patients was managed by orthodontists at our hospital from the early stage and treated by maxillary expansion during the mixed dentition period. None of the patients underwent surgical maxillary expansion after reaching skeletal maturity in adulthood. Dental implants (Brånemark system Mk III; Nobel Biocare Services AG, Zürich, Switzerland) were placed at sites of bone grafting in the alveolar cleft region. In addition, the retainer, which had been worn since the end of orthodontic treatment, was removed after implant treatment.

Mean age at bone graft to the alveolar cleft region was 24.4 years (range, 10–39 years). In these patients, orthodontic treatment was performed while securing a space for the placement of implants with a retainer, and implant surgery was performed under local anesthesia after the completion of orthodontic treatment. Mean age at implant surgery was 29.4 years (range, 19–44 years). Mean time to implant surgery after bone grafting was 43 months (range, 5–188 months). The number of implants placed in alveolar clefts was 1 in 9 patients, 2 in 7 patients, and 3 in 4 patients. Mean duration of follow‐up from CT immediately before implant surgery to CT after completion of implant treatment was 38 months (range, 7–102 months). The number of implants placed in alveolar clefts was 1 in 9 patients (UCP, *n* = 7; BCLP, *n* = 2), 2 in 7 patients (UCLA, *n* = 1; UCP, *n* = 4; BCLP, *n* = 2), and 3 in 4 patients (BCLP, *n* = 4). Implants placed in the alveolar cleft region demonstrated osseointegration in all patients without peri‐implantitis‐induced abnormal bone resorption around the implants or implant mobility and removal.

### 3.2 Measurement results

#### 3.2.1 Results from analysis of measurement accuracy

Population means of the 1st, 2nd, and 3rd measured values for W3, W4, W5, and W6 were comparable before and after surgery, and no significant difference was noted.

#### 3.2.2 DAW in each measurement region

Minimum and maximum W3 values before implant treatment were 20.53 and 27.15 mm, respectively, while those of W4 were 24.54 and 33.91 mm, respectively, those of W5 were 27.58 and 38.69 mm, respectively, and those of W6 were 31.14 and 40.85 mm, respectively. Minimum and maximum W3 values after completion of implant treatment were 19.72 and 26.21 mm, respectively, those of W4 were 25.36 and 33.24 mm, respectively, those of W5 were 26.27 and 39.95 mm, respectively, and those of W6 were 32.63 and 41.25 mm, respectively.

Mean difference in DAW after treatment in each measurement region was −0.58 mm in the canine region, −0.05 mm in the first premolar region, −0.11 mm in the second premolar region, and 0.51 mm in the first molar region (Table 1).

**TABLE 1:** Width and changes in dental arch for participants with cleft and lip palate

	BIT (mm)			AIT (mm)			AIT‐BIT (mm)	
	mean ± SEM	Min	Max	mean ± SEM	Min	Max	mean ± SEM	*p*‐value
13–23	24.02 ± 0.49	20.53	27.15	23.14 ± 0.59	19.72	26.21	−0.58 ± 0.21	0.103
14–24	28.29 ± 0.56	24.54	33.91	28.10 ± 0.55	25.36	33.24	−0.05 ± 0.27	0.106
15–25	33.35 ± 0.64	27.58	38.69	33.25 ± 0.72	26.27	39.95	−0.11 ± 0.13	0.394
16–26	36.60 ± 0.70	31.14	40.85	37.15 ± 0.60	32.63	41.25	0.51 ± 0.26	0.033

Abbreviations: AIT, after implant treatment; BIT, before implant treatment; SEM, standard error of the mean.

#### 3.2.3 Results from statistical analysis of changes in DAW

The significance level, as determined by the Wilcoxon signed‐rank test, was *p* = 0.121 in the canine region, *p* = 0.1055 in the first premolar region, and *p* = 0.394 in the second premolar region. Thus, no significant differences in values after treatment were identified. On the other hand, a significant difference in W6 was seen after treatment (*p* = 0.033; Table 1).

#### 3.2.4 Results from analysis of factors influencing differences in DAW after treatment

Factors influencing W6, which showed a significant difference after treatment, were investigated by ANCOVA, with the absolute value of the mean change after treatment (interdental width before treatment ‐ interdental width after treatment) regarded as the response variable and the following six items regarded as explanatory variables: sex; cleft type; age at bone graft to the alveolar cleft region; time to implant treatment after bone grafting; number of implants; and duration of follow‐up.

First, the correlation coefficient between explanatory variables was determined and the absence of a combination of variables with a large correlation coefficient causing multicollinearity was confirmed. ANCOVA of the difference in W6 after treatment was then performed using a statistical model that included all explanatory variables. Akaike's information criterion (AIC) (Crawley, 2005) was used as the judgment criterion for the ANCOVA model formula. We searched for the optimal model formula that acquired the minimum AIC using the step function of R and the following regression equation was acquired:

B–A = −0.6206 × S + 1.0209 × C–0.8438 × I–0.4966 (B: W6 before treatment; A: W6 after treatment; S: sex; C: cleft type; I: number of implants).

This equation did not reject the null hypothesis of sex, that the regression coefficient would be 0 (*p* = 0.614). The null hypothesis was rejected for the following variables: C, cleft type (*p* = 0.016); and I, number of implants (*p* = 0.037).

Taken together, the difference in DAW in the first molar region after treatment increased in the order of BCLP, UCLP, and CLA, and the difference was slightly decreased in patients with larger numbers of implants.

## 4 DISCUSSION

The purpose of performing secondary bone graft to the alveolar cleft during the mixed stage of dentition is to allow sufficient oral rehabilitation by inducing canine tooth eruption or orthodontic movement of permanent teeth in the cleft region without prosthodontic treatment. However, construction of occlusion by orthodontic treatment alone is difficult in some cases due to various reasons, such as missing teeth and aplasia or dysplasia of the teeth in the area of the alveolar cleft. Implant treatment in these cases is considered an excellent treatment option, since cutting of the adjacent tooth (e.g., fixed bridge) and irreversible stress are avoided. Matsui et al. (2006) reported that the implant survival rate in CLP patients who received a bone bridge formed by secondary bone graft to the alveolar cleft was 98.6% after ≥5 years of follow‐up. Wang et al. (2014) collected and analyzed previous clinical studies, and reported that the implant survival rate in cases involving alveolar clefts was 91.5% after 54.3 months of follow‐up. Wermker et al. (2014) reported an implant survival rate in alveolar clefts of 94.3% after 34 months of follow‐up. Sales et al. (2019) reviewed previous studies using an electronic database and showed an implant survival rate of 93% in alveolar clefts after 60.5 months of follow‐up. These reports suggest that implants placed in the bone graft region of clefts have a survival rate almost equivalent to that of implants placed in the jaw bone of non‐cleft patients.

Orthodontic treatment of CLP patients has often involved transverse and sagittal expansion of the dental arches, since maxillary retrusion with anterior and lateral crossbites is a common finding (Mars et al., 1992). As implant treatment does not prevent relapse of the DAM, unlike a fixed prosthesis (Caballero et al., 2019), the DAM may lack stability after treatment. Thus, in the present study, DAM stability was evaluated before and after implant treatment of the alveolar cleft region by measuring DAW of the upper jaw on CT images acquired before and after surgery. The dental cusps on the buccal side were used as the measurement points for DAW in previous studies (Anttila et al., 2004; Berger et al., 1998; Marcusson & Paulin, 2004), but the present study included patients in whom artifacts were generated by coronal restorations, making CT images of the coronal region unclear. Accurately identifying the coronal cusps was thus difficult. Measurement points were set at the canine cusps and the cemento‐enamel junctions right below the mesiolingual cusps of the first and second premolars and first molar.

The presence of changes in DAM was analyzed by measuring W3, W4, W5, and W6 on CT images acquired before and after implant treatment, and differences in width after treatment were calculated. The mean difference after treatment was less than 1.0 mm in all widths, and only W6 showed a significant difference after treatment. This suggests that changes in DAW after implant treatment were small, possibly because the maxilla was expanded by orthodontic treatment during the mixed dentition period, and a stable dental arch was acquired. Expansion by orthodontic treatment during the mixed dentition period is effective in improving dental arch constriction (Sari et al., 2003), and has been reported to achieve stable results until permanent dentition (Mutinelli et al., 2015).

A significant difference was noted in W6 after treatment, but the mean difference was 0.63 mm, suggesting quote limited clinical significance. However, when an ANOCVA analysis of the six factors regarded as explanatory variables was performed to identify factors associated with changes in W6 after treatment, a significant difference was noted in cleft type and the number of embedded implants. This clarified that sex, age at bone graft, time to implant treatment after bone grafting, and duration of follow‐up do not influence W6. The difference in W6 increased in the order of BCLP, UCLP, and CLA, and decreased as the number of implants increased. In patients with CLP, the dental arch narrows in the molar region due to the influence of scar tissue that forms after palatoplasty. The presence of the largest difference in W6 in BCLP may thus have been due to a stronger influence of scar tissue in comparison to other cleft types, and the smallest influence noted in cleft lip and alveolus may have been the presence of the least amount of scar tissue in the palatal region. This may also have been because the DAM was close to normal morphology after an appropriate number of implants was planted in an appropriate interdental space, which was secured by orthodontic treatment, in patients with multiple implants, and attachment of a retainer before implant treatment stabilized the DAM. To maintain the DAM after implant treatment, confirming that the DAM after orthodontic treatment was stable before implant treatment was considered important. The population of the present study was relatively small. To clarify factors influencing changes in DAM after implant treatment in CLP patients, analysis of a larger cohort may be necessary.

Fixed partial dentures may lead to stabilization of the dental arches (Caballero et al., 2019), but recession was noted and the DAW changed even when the dentition was permanently fixed using a fixed bridge (Ramstad & Jendal, 1997). Moreover, the maxillary dimensions were not stabilized after orthodontic and implant‐supported prostheses in CLP patients (Pucciarelli et al., 2020). The maxillary DAM after dental implant placement in the alveolar cleft and its actual state remain unclear. Consideration of recession‐induced changes in DAW is thus necessary, even when implant treatment is applied to the bone bridge formed in the alveolar cleft by the secondary bone graft. The present study analyzed changes in DAM by evaluating W3, W4, W5, and W6 on CT images acquired before and after implant treatment in CLP patients who underwent implant treatment of the alveolar cleft region. Although the study population was relatively small, the mean difference in DAW after treatment was ≤1 mm in all measured regions, and changes in DAW were slight.

Although CLP has been reported to cause stenosis of the dental arch due to scarring from palate surgery, the present study suggested that implant treatment of the alveolar cleft region results in a slight reduction in DAW after the completion of treatment. Pucciarelli et al. reported instability in inter‐canine measurement of the cleft area in a CLP patient with orthodontic and implant treatment (Pucciarelli et al., 2020). Meanwhile, no significant differences in W3 were seen between before and after treatment in our study. Since all patients underwent maxillary expansion during the mixed dentition period in our study, maxillary expansion in the early stage and bone graft were suggested to provide stability to the DAM in CLP patients with implant treatment.

In future studies, accumulation of more patients will be necessary to clarify factors influencing DAM in CLP patients who undergo implant treatment in the alveolar cleft region.

## CONFLICT OF INTEREST

The authors have nothing to disclose.

## AUTHOR CONTRIBUTIONS

Conception and design: Shirota T, Sato H and Ahn K. Acquisition of data: Shirota T, Sato H and Ahn K. Analysis and interpretation of data: Ahn K, Kurihara Y and Ogura H. Drafting the manuscript: Ahn K, Sato H and Shirota T. Revising the manuscript for intellectual content: Sato H, Ogura Y and Shirota T. Final approval of the completed manuscript: Ahn K, Sato H, Kurihara Y, Ogura H, Shirota T.

## Data Availability

The datasets analyzed during the current study available from the corresponding author on reasonable request.
